# Unveiling the genetic architecture and transmission dynamics of a novel multidrug-resistant plasmid harboring *bla*_*NDM-5*_ in *E. Coli* ST167: implications for antibiotic resistance management

**DOI:** 10.1186/s12866-024-03333-1

**Published:** 2024-05-23

**Authors:** Dengke Han, Suzhen Ma, Chenhong He, Yuxing Yang, Peng Li, Lanfen Lu

**Affiliations:** 1 Department of Laboratory Medicine, Zhongshan City People’s Hospital, Zhongshan, 528403 Guangdong China; 2 Department of Emergency, Zhongshan City People’s Hospital, Zhongshan, 528403 Guangdong China; 3 Chinese PLA Center for Disease Control and Prevention, 20 DongDa Street, Fengtai District, Beijing, 100071 China

**Keywords:** Multidrug-resistant *Escherichia coli*, Carbapenems resistance, *bla*_NDM-5_ gene, IncF plasmid, ST167

## Abstract

**Background:**

The emergence of multidrug-resistant (MDR) *Escherichia coli* strains poses significant challenges in clinical settings, particularly when these strains harbor New Delhi metallo-ß-lactamase (NDM) gene, which confer resistance to carbapenems, a critical class of last-resort antibiotics. This study investigates the genetic characteristics and implications of a novel *bla*_*NDM-5*_-carrying plasmid pNDM-5-0083 isolated from an *E. coli* strain GZ04-0083 from clinical specimen in Zhongshan, China.

**Results:**

Phenotypic and genotypic evaluations confirmed that the *E. coli* ST167 strain GZ04-0083 is a multidrug-resistant organism, showing resistance to diverse classes of antibiotics including ß-lactams, carbapenems, fluoroquinolones, aminoglycosides, and sulfonamides, while maintaining susceptibility to monobactams. Investigations involving S1 pulsed-field gel electrophoresis, Southern blot analysis, and conjugation experiments, alongside genomic sequencing, confirmed the presence of the *bla*_*NDM-5*_ gene within a 146-kb IncFIB plasmid pNDM-5-0083. This evidence underscores a significant risk for the horizontal transfer of resistance genes among bacterial populations. Detailed annotations of genetic elements—such as resistance genes, transposons, and insertion sequences—and comparative BLAST analyses with other *bla*_*NDM-5*_-carrying plasmids, revealed a unique architectural configuration in the pNDM-5-0083. The MDR region of this plasmid shares a conserved gene arrangement (*repA-IS15DIV-bla*_*NDM-5*_*-ble*_*MBL*_*-IS91-suI2-aadA2-dfrA12*) with three previously reported plasmids, indicating a potential for dynamic genetic recombination and evolution within the MDR region. Additionally, the integration of virulence factors, including the *iro* and *sit* gene clusters and *enolase*, into its genetic architecture poses further therapeutic challenges by enhancing the strain’s pathogenicity through improved host tissue colonization, immune evasion, and increased infection severity.

**Conclusions:**

The detailed identification and characterization of pNDM-5-0083 enhance our understanding of the mechanisms facilitating the spread of carbapenem resistance. This study illuminates the intricate interplay among various genetic elements within the novel *bla*_*NDM-5*_-carrying plasmid, which are crucial for the stability and mobility of resistance genes across bacterial populations. These insights highlight the urgent need for ongoing surveillance and the development of effective strategies to curb the proliferation of antibiotic resistance.

**Supplementary Information:**

The online version contains supplementary material available at 10.1186/s12866-024-03333-1.

## Background

Multi-drug-resistance (MDR) bacterial pathogens have been increasing worldwide and is now considered a significant public health threat. Several recent studies have documented the emergence of MDR *Enterobacteriaceae* from various sources [1–7], underscoring the need for proper antibiotic use. Besides, routine antimicrobial susceptibility testing is crucial to determine the most effective antibiotic treatments and to monitor the spread of emerging MDR strains. According to recent surveillance, the World Health Organization (WHO) has ranked carbapenemase-producing *Enterobacteriaceae* (CPE) as the third MDR bacteria in priority level one [8, 9], due to their ability to resist carbapenems which considered the last line of defense against MDR infections [4, 10]. These pathogens, including prominent species like *Klebsiella pneumoniae* and *Escherichia coli* (*E. coli*), have shown a disturbing increase in prevalence across various regions worldwide [11, 12]. This surge is largely driven by the acquisition and dissemination of carbapenemase genes such as *bla*_*KPC*_, *bla*_*NDM*_, and *bla*_*OXA*_ [13, 14], which confer high-level resistance to nearly all ß-lactams, including carbapenems.

To date, 24 variants of the New Delhi metallo-ß-lactamase (NDM) gene have been identified in bacteria harboring *bla*_*NDM*_ across the globe [13, 15–17], in which the NDM-5 is one of the most common variants encountered among *Enterobacteriaceae* with highly restricted availability of sensitive antibiotics [18]. Although reports of CPE carrying *bla*_NDM-5_ from various regions have attracted widespread attention [18–20], its genetic backgrounds are highly conserved and often located on mobile genetic elements as well as virulence factors that facilitate their spread [21].

Mobile elements such as insertion sequences and transposons are pivotal in the homologous recombination processes that boost the adaptability and survival of CPE. These genetic elements facilitate the acquisition and dissemination of antimicrobial resistance genes and contribute to the structural rearrangement of plasmids, impacting the fitness and virulence of bacterial pathogens [22, 23]. For example, insertion sequences like *IS26* can initiate the formation of composite transposons that harbor multiple resistance genes, complicating the treatment of infections caused by these bacteria [24, 25]. Additionally, the incorporation of toxin-antitoxin systems and virulence factors, such as the *pemK*/*pemI*, *iro*, and *sit* gene clusters, into these plasmids enhances the pathogenic potential of bacteria. These elements help bacteria more effectively colonize host tissues, evade immune responses, and increase the severity of infections [26–29]. Otherwise, both resistance and virulence genes play crucial roles in the formation of biofilms, which significantly contribute to bacterial resistance to antibiotics through multiple mechanisms, including limited antibiotic penetration, nutrient limitation, slow growth, and adaptive stress responses [30, 31]. Therefore, studying the mobile genetic elements and virulence factors within plasmids provides crucial insights that can help prevent and treat infections associated with clinical multidrug-resistant strains harboring the *bla*_*NDM-5*_.

This study aims to thoroughly characterize a MDR plasmid harboring the *bla*_*NDM-5*_ gene in an *E. coli* isolate, emphasizing the elucidation of its genetic architecture and the mechanisms underlying the spread of this resistance factor. Specifically, the research focuses on analyzing the origins and consequences of homologous recombination within the MDR region of the plasmid, as well as the roles played by mobile genetic elements and virulence factors in the dissemination of resistance. By gaining a deeper understanding of these mechanisms, this study seeks to contribute substantially to the development of more effective strategies for managing and controlling the spread of CPE infections, thereby addressing an urgent need in the field of antibiotic resistance management.

## Materials and methods

### Isolation and identification of strain GZ04-0083

Strain GZ04-0083 was found in a stool specimen collected from a 37-year-old male patient with renal insufficiency who had been undergoing peritoneal dialysis for over five years. The patient was admitted to the intensive care unit (ICU) of Zhongshan City People’s Hospital in Zhongshan, Guangdong Province of China due to dialysis-related peritonitis, fever, and severe diarrhea. Fecal swabs were inoculated onto blood agar plates (KMJ, Shanghai, China) and incubated at 37? overnight to promote growth. Using sterile loop, single colony was streaked onto MacConkey agar (CRmicrobio, Jiangmen, China) and incubated at 37? for 18–24 h to isolate gram-negative bacteria as described by Kelly. M. T et al. [32]. Suspected *E. coli* colonies were adjusted to a 0.5 McFarland standard for consistency and subsequently confirmed through biochemical assays using the Vitek-2 compact system (bioMérieux, Marcy-l’Étoile, France). This system evaluates various enzymatic activities characteristic of *E. coli*, following both the manufacturer’s guidelines and the established clinical procedures detailed by Perez-Vazquez, M [33]. Ethical approval for this study was obtained from the Clinical Research and Laboratory Animal Ethics Committee of Zhongshan People’s Hospital (approval #K2022-008).

### Antimicrobial susceptibility test

The antimicrobial susceptibility was assessed using the Vitek-2 compact system and minimum inhibitory concentration (MIC) test strips. *E. coli* ATCC 25,922 served as the control strain, with result analysis adhering to Clinical and Laboratory Standards Institute (CLSI) guidelines [34]. Briefly, colonies confirmed as *E. coli*, along with the control strain, were standardized to a 0.5 McFarland turbidity. This suspension was further diluted with 3 ml of 0.45% NaCl and 145 µL of bacterial suspension. The diluted sample was then processed using the AST-N334 card in the Vitek-2 system, according to the manufacturer’s instructions.

MIC values for various antibiotic classes, including ß-lactams, carbapenems, fluoroquinolones, and aminoglycosides as listed in Table 1, were determined using MTS. Briefly, the bacterial suspension was evenly spread on a Mueller-Hinton agar plate (Detgerm, Guangzhou, China), and MIC test strips (MTS, Liofilchem, Italy) were carefully placed on the agar surface. After incubating at 37 °C for 16–20 h, MIC values were read where the edge of the inhibition zone met the scale on the test strips. Results were categorized as susceptible (S), intermediate (I), or resistant (R). The isolates were classified as MDR, characterized by resistance to at least one agent in three or more antibiotic classes as defined by Magiorakos et al. [35]. The Multiple Antibiotic Resistance (MAR) Index was calculated by dividing the number of antibiotics to which the organism shows resistance by the total number of antibiotics tested, in accordance with Pauls et al. description [36] and CLSI guidelines.

**Table 1:** Antibiotic susceptibility results of *E. coli* GZ04-0083 isolate

Antibiotic classes	Antibiotic name	MIC (µg/ml)	Interpretive category	Resistance genes
ß-lactams	Ampicillin (AMP)	= 64	R	*bla*_*TEM-1B*_^a^
	Piperacillin (PIP)	128	R	
	Cefepime (FEP)	= 64	R	
	Ceftriaxone (CRO)	= 64	R	
	Ceftazidime (CAZ)	= 64	R	
	Cefotetan (CTT)	= 64	R	
	Cefazolin (CZO)	= 64	R	
ß-lactams with ß-lactamase inhibitors	Ampicillin/sulbactam (SAM)	= 32	R	*bla*_*TEM-1B*_^a^
	Piperacillin/tazobactam (TZP)	128	R	
Monobactam	Aztreonam (ATM)	2	S	-
Carbapenems	Ertapenem (ETP)	= 8	R	*bla*_*NDM-5*_^a^
	Imipenem (IMP)	= 16	R	
Fluoroquinolones	Ciprofloxacin (CIP)	= 4.0	R	*gyrA, parC*^b^
	Levofloxacin (LVX)	= 8.0	R	
Aminoglycosides	Amikacin (AK)	= 64	R	*aadA2, rmtB*^a^
	Tobramycin (TOB)	= 16	R	
	Gentamicin (GEN)	= 16	R	
Sulfonamide	Trimethoprim/sulfamethoxazole (SXT)	= 320	R	*suI1, drfA2*^a^
Nitrofuran	Nitrofurantoin (NIT)	64	I	*nfsA, nfsB*^b^

*MIC* minimum inhibitory concentration, *S* susceptible, *I* intermediate, *R* resistant

^a^Antimicrobial-resistance genes identified in plasmid annotation

^b^Antimicrobial-resistance genes identified in chromosome annotation

### S1 pulsed-field gel electrophoresis, southern blot, and conjugation experiment

S1 endonuclease (Takara, Dalian, China) was used to analyze the bacterial genomic DNA of strain GZ04-0083. Pulsed-field gel electrophoresis (PFGE) was performed in a CHEF-DRIII system (Bio-Rad, Hercules, USA) to isolate DNA fragments and analyze the band patterns to obtain PFGE profiles at 6 V/cm with an initial pulse time of 0.22 s and a final pulse time of 26.29 s for 15 h. The separated plasmid DNA was transferred to a 0.45 µm positively charged nylon membrane (Solabio, Beijing, China) and hybridized with a digoxigenin-labeled DNA probe specific to the *bla*_NDM-5_ gene. The southern blot experiment was performed according to the manufacturer’s manual of the DIG High Prime DNA Labeling and Detection Start Kit I (Roche, Indianapolis, USA).

A conjugation experiment was conducted to assess the transferability of the resistance plasmids. The experiment was conducted with GZ04-0083 as donors and the sodium-azide-resistant *E. Coli J53* as recipient. Firstly, GZ04-0083 was mixed with J53 at a ratio of 4:1 using a filter (0.22-µm pore size) mating assay, and the filter was incubated on a BHI agar (KMJ, Shanghai, China) at 37 °C overnight. Secondly, the transconjugants were selected on BHI agar plates supplemented with 4 mg/ml meropenem and 200 mg/ml sodium azide after 72 h of incubation, and the conjugation frequency was calculated as the ratio of transconjugants to recipient cells. Lastly, the plasmids in transconjugants were also confirmed by S1-PFGE.

### Plasmid sequencing and bioinformatics analysis

The whole genomic DNA of GZ04-0083 was extracted from cultured bacteria using the High Pure PCR Template Preparation Kit (Roche, Basel, Switzerland), following the manufacturer’s instructions. Second-generation and nanopore sequencing was performed through the Illumina MiSeq sequencing platform and the Oxford Nanopore MinION sequencer, respectively.

The chromosome and plasmids were assembled using Unicycler (v0.4.8) to yield the complete gene sequences [37]. Seven housekeeping genes (*adk*, *fumC*, *gyrB*, *icd*, *mdh*, *purA*, and *recA*) of *E. Coli GZ04-0083* were matched by the MLST website server (https://mlst.warwick.ac.uk/mlst/dbs/Ecoli) to obtain the sequence number of each allele and identify the multi-locus sequence typing of the strain [38].

The genomic sequences of the plasmids were annotated using the RAST Server and Protein BLAST (https://blast.ncbi.nlm.nih.gov/Blast.cgi) [39, 40], and the plasmids were indexed with the ISFinder database (https://www-is.biotoul.fr/) [41] for transposons, insertion sequences, and other structures in the genetic environment were identified. Plasmid Finder was used to identify the plasmids for the replication type (https://cge.cbs.dtu.dk/services/PlasmidFinder) [42]. The virulence-related genes in bacterial and plasmid genomes were identified by VFDB Blast (http://www.mgc.ac.cn/VFs) [43]. Inkscape 1.0 was used to map the overall structure of the plasmids, the fine structure comparison of the multi-resistant regions, and the near-source linear structural comparison of the multi-resistant regions of the plasmids. Sequence comparison and map generation were performed using BLAST (http://blast.ncbi.nlm.nih.gov) and Easyfig (version 2.1), respectively [44].

## Results

### Phenotypic multidrug resistance of ***E. coli ***Strain GZ04-0083

To present the phenotypic characteristics and antimicrobial resistance profile of the *E. coli* strain GZ04-0083 recovered from a clinical sample, antimicrobial susceptibility test was performed using both AST-N334 susceptibility card read by Vitek-2 and MIC test strips. In the analysis of 19 antibiotics, the *E. coli* strain GZ04-0083 exhibited resistance to a variety of commonly used antibiotics across different classes. It was resistant to ß-lactams, including ampicillin and ceftriaxone; carbapenems, such as ertapenem and imipenem; fluoroquinolones, including ciprofloxacin and levofloxacin; and aminoglycosides like amikacin and gentamicin. In contrast, the strain remained susceptible to the monobactam antibiotic aztreonam and exhibited intermediate resistance to nitrofurantoin. The detailed resistance profile is presented in Table 1. According to the criteria established by Magiorakos et al., strain GZ04-0083 is classified as a MDR strain, with the MAR index of 0.89.

### Identification of a ***bla***_***NDM-5 ***_Carrying Plasmid in GZ04-0083 isolate

**Fig. 1:**
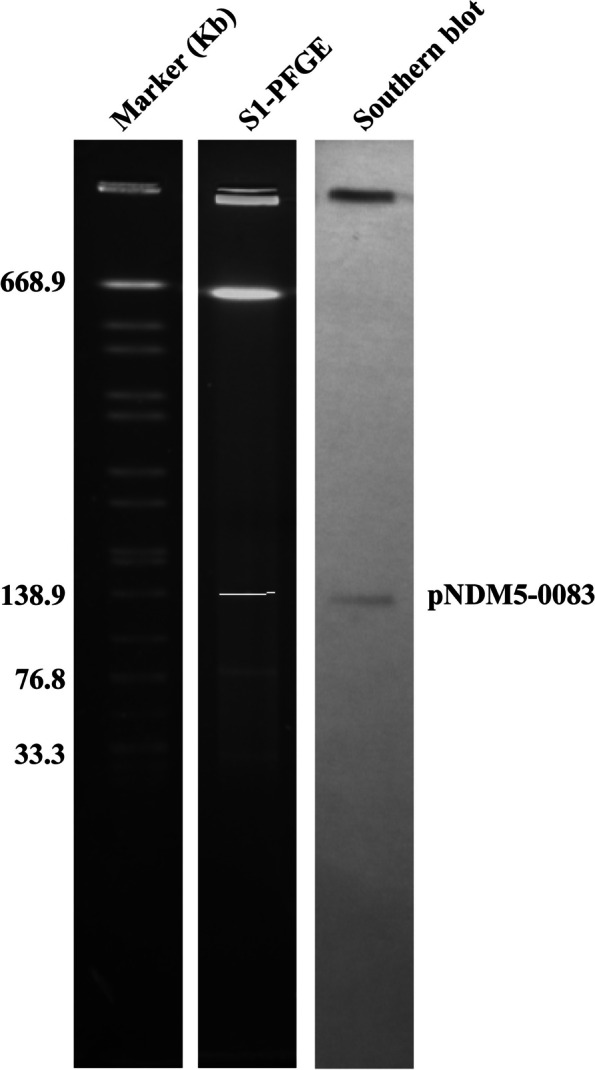
S1-PFGE and Southern blot of strain GZ04-0083. The left lane is the marker of the S1-PFGE profile of the reference strain; the middle lane of the S1-PFGE is a result of strain GZ04-0083 S1 enzymatically cleaved plasmid DNA; the right lane is southern blot hybridization of the *bla*_*NDM-5*_-specific probe

The antimicrobial susceptibility testing indicated that the GZ04-0083 isolate might resist carbapenems by producing ß-lactamase. To determine the genetic basis of carbapenem resistance in the GZ04-0083 isolate and assess its potential for horizontal gene transfer among *Enterobacteriaceae*, the S1-PGFE analysis revealed the presence of three plasmids (about 140, 80, and 30 kb, respectively) in GZ04-0083, and the *bla*_NDM-5_ gene was found in the 140-kb plasmid by southern blot (Fig. 1and Supplementary Figs. 1 and 2). Further conjugation experiment suggested that the *bla*_NDM-5_-carrying plasmid was able to transfer from the donor strain GZ04-0083 to the recipient *E. coli* J53 strain with meropenem and sodium azide as co-selection markers, and the conjugation frequency was 3.38 ± 0.82 × 10^-5^ per recipient. Accordingly, the GZ04-0083 isolate demonstrated multidrug resistance characteristics, evidenced by its resistance to a broad spectrum of antibiotics, including carbapenems, and the presence of the *bla*_NDM-5_ gene on a 140-kb plasmid capable of conjugative transfer to other *Enterobacteriaceae*.

#### Genome sequencing results confirming the presence of the NDM resistance gene

Whole-genome sequencing was conducted to validate the plasmid data and ascertain the molecular type of the *E. coli* GZ04-0083 strain. By assembling both short Illumina reads and long PromethION reads with Unicycler (v0.4.8), we established that the GZ04-0083 strain’s genome consists of a single chromosome (4.91 Mb) and three plasmids (146, 89, and 27 kb). MLST analysis classified the GZ04-0083 isolate as ST167, the predominant clone among NDM-producing *E. coli* strains in China. A BLAST alignment of *bla*_NDM-1_ and *bla*_NDM-5_ with the GZ04-0083 nucleotide sequence confirmed the presence of the *bla*_NDM-5_ gene within the 146-kb plasmid, aligning with the Southern blot findings presented in Fig. 1. A further plasmid MLST analysis identified the 14-kb *bla*_NDM-5_-carrying plasmid as an IncFIB-type plasmid with a linear topology, which was designated as pNDM-5-0083.

### Genotypic multidrug resistance of ***E. coli ***Strain GZ04-0083

To explore the relationship between phenotypic resistance and genotypic features in the GZ04-0083 isolate, our analysis extended beyond the identification of the *bla*_*NDM-5*_-carrying plasmid. Table 1 demonstrates that the *E. coli* GZ04-0083 isolate exhibits a robust multidrug-resistant profile, supported by both phenotypic and genotypic evidence. Chromosomally encoded genes such as *gyrA* and *parC* are associated with fluoroquinolone resistance [45], while *nfsA* and *nfsB* contribute to decreased susceptibility to nitrofurantoin [46], underlying the strain’s inherent resistance mechanisms. Moreover, the pNDM-5-0083 plasmid harbors additional resistance genes, enhancing the isolate’s capability to withstand various antibiotics. These genes include *bla*_*NDM-5*_, which confers resistance to carbapenems, and *bla*_*TEM-1B*_, *dfrA12*, *sul1*, *aadA2*, and *rmtB*, which collectively provide resistance against ß-lactams, sulfonamides, trimethoprim, and aminoglycosides.

### Genetic characterization of pNDM-5-0083 as a novel ***bla***_***NDM-5***_-carrying plasmid

**Fig. 2:**
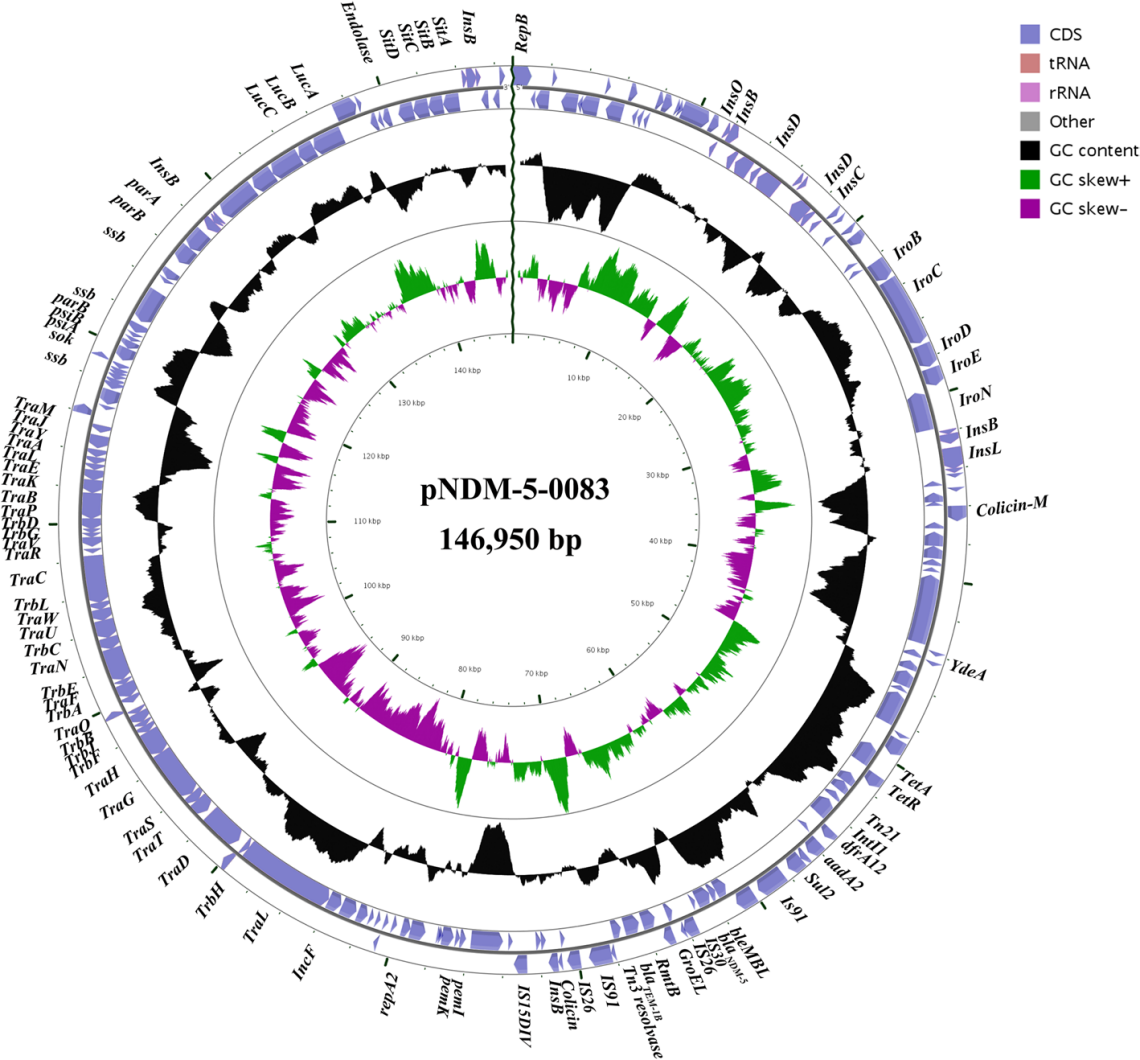
Gene structure of ***E. Coli ***GZ04-0083 carrying plasmid pNDM-5-0083. The outer ring represents the annotation of the plasmid, and the genes are annotated with different colors according to their functions. Yellow represents transfer RNA (TransferRNA, tRNA); red represents ribosomal RNA (rRNA); blue-purple CDS (CondingSequence) is in mRNA, protein-coding region; black GC Content represents the content of the plasmid; GC Shew represents plasmid bias and measures the G versus C content of single-stranded DNA in the plasmid: Green GC Shew + represents plasmid single-stranded DNA with greater G content than C. Purple GC Shew- represents plasmid single-stranded DNA with less G content than C. Red frame represents pNDM-5-0083 plasmid carrying resistance genes

Studying the plasmid’s backbone and its array of mobile genetic elements and virulence factors will help elucidate the mechanisms underlying the propagation of resistance traits. Consequently, using ISFinder, PlasmidFinder and VFDB Blast for annotation, several key elements were identified in the plasmid, especially in the MDR region (Fig. 2**)**. The backbone region of pNDM-5-0083 included a replication protein (*repA*), stabilization proteins (*parA* and *parB*), a conjugative splice transfer system of plasmids (*traABCDEFGHIJKLMNPQRSTUVWY* and *trbABCDEFGIJ*) and iron transporters as virulence factors (*iroBCDEN*, *lucABC*, and *sitABCD*). Within and surrounding the MDR region, the plasmid harbored another replication protein (*repA2*), resistance genes (*bla*_NDM-5_, *bla*_TEM-1B_, *suI1, dfrA12, tetA, aadA2*, and *rmtB*), transposon elements (*intI1*, *Tn21*, and *Tn3*), insertion sequences (*IS15DIV*, *IS26*, *IS30*, *IS91*, and *insBCDOL*) and a chaperone protein (*GroEL*) which are associated with resistance genes transmission. In addition, the pNDM-5-0083 plasmid also harbors a toxin-antitoxin system (*pemK/pemI*) and other virulence genes, including *colicin-M* and *enolase*.

**Fig. 3:**
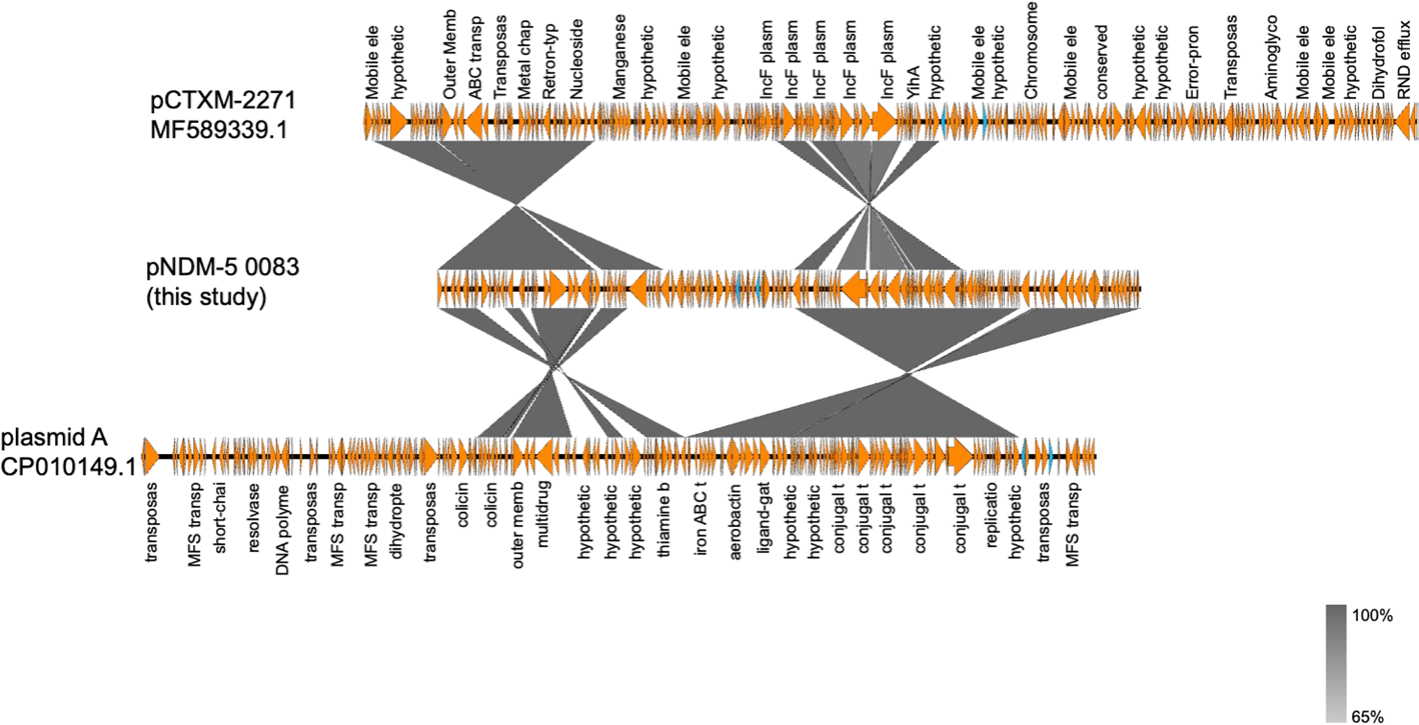
Genomic comparison of pNDM-5-0083 with pCTXM-2271 (GenBank accession number MF589339.1) as well as the plasmid A plasmid (GenBank accession number CP010149.1). The blue color indicates resistance genes, and each arrow represents one gene. The depth of grey shading indicates the similarity of each part of the genome with the sequence of the pNDM-5-0083 genome. The darker the shade of grey, the higher the degree of similarity. The graph shows fragments with at least 65% similarity, so fragments below this threshold are not shown

To clarify whether the pNDM-5-0083 had fragments of different evolutionary origins, the pNDM-5-0083 plasmid was BLAST-matched in the NCBI database to screen for plasmids with homology to the pNDM-5-0083 plasmid (Fig. 3). The results revealed that pNDM-5-0083 shared a 99.89% identity with 79% query coverage to pCTXM-2271 plasmid (GenBank accession number MF589339.1) which is known MDR plasmid isolated from *E. coli* strain 2271 [47], and a 99.96% identity with 83% query coverage to plasmid A (CP010149.1) which was identified in *E. coli* strain D6 from dog. The high sequence identities but partial query coverages, particularly in the MDR region, indicate that pNDM-5-0083, while closely related to these known plasmids, likely contains unique genetic elements do not present in the aligned plasmids. This partial alignment suggests that pNDM-5-0083 may harbor unique sequences that contribute to its unique resistance profile, meriting further investigation to fully understand its role in antimicrobial resistance.

## Comparative analysis of the MDR Region in pNDM-5-0083

**Fig. 4:**
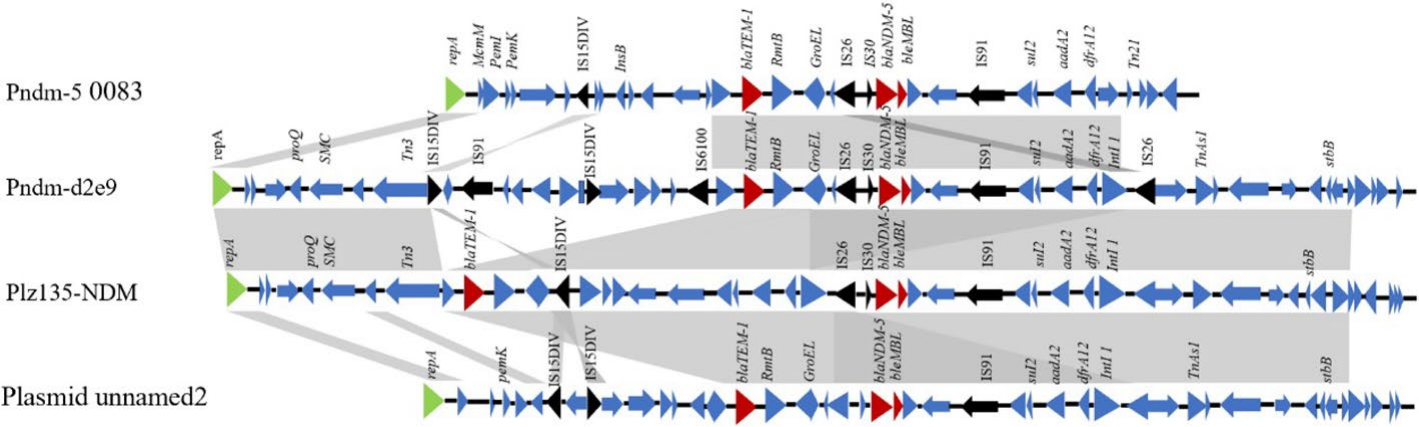
Comparison of the MDR region gene clusters of pNDM-5-0083, pNDM-d2e9, pLZ135-NDM, and Plasmid unnamed2. Arrows indicate open reading frames (ORFs) and transcriptional direction. Green arrows indicate the replicons (repA encodes the replication initiation protein). Black arrows indicate the insertion sequence (IS) of mobile elements. Red arrows indicate drug resistance genes (*bla*_*NDM-5*_, *bla*_*TEM-1B*_, and *ble*_*MBL*_). Blue arrows indicate stability-related genes and hypothetical protein-coding genes. Grey shading indicates regions of high homology between the four plasmids. Plasmid pNDM-d2e9 is derived from the *Escherichia coli* ECONIH6 strain. Plasmid pLZ135-NDM is derived from *E. coli*. Plasmid unnamed2 is derived from *E. coli* strain FDAARGOS_433

To elucidate the evolutionary dynamics of the MDR region in the pNDM-5-0083 plasmid, we profiled gene clusters analogous to the MDR region of pNDM-5-0083 and constructed a linear analysis of these gene clusters. As shown in Fig. 4, the analysis revealed that the MDR region of pNDM-5-0083 encompasses seven drug resistance genes: *bla*_NDM-5_, *bla*_TEM-1B_, *ble*_*MBL*_, *sul2, aadA2*, and *drfA12*. BLAST comparison showed high similarity between the MDR region of pNDM-5-0083 and those of pNDM-d2e9 (CP026201.1), pLZ135-NDM (MF353156.1), and Plasmid unnamed2 (CP104348.1), which are associated with CPE isolates. These plasmids share a conserved gene arrangement: *repA-IS15DIV-bla*_NDM-5_*-ble*_*MBL*_*-IS91-suI2-addA2-dfrA12*, featuring identical resistance genes and insertion sequences. This consistent arrangement highlights a common evolutionary origin and suggests a mechanism for the persistence and propagation of resistance traits among these plasmids.

## Discussion

In recent years, *bla*_NDM-5_*-*producing *E. coli* ST167 has been reported across various countries and regions globally [48–52]. Researchers indicates that the *bla*_NDM-5_ resistance gene in *E. coli* tends to spread through a plasmid-mediated horizontal transmission [53], significantly contributing to the rapid proliferation of this resistance gene among *E. coli* population. Whole-genome sequencing analysis can enhance our understanding of the mechanisms behind the spread of *bla*_NDM-5_, providing detailed insights into the mobile genetic elements that facilitate this transmission and the evolutionary pressures that drive the dissemination of resistance across different environments [54].

The pNDM-5-0083 plasmid harbored seven resistance genes, including *bla*_NDM-5_, *bla*_TEM-1B_, *dfrA12*, *suI1*, *tet(A)*, *aadA2*, and *rmtB*, which has similarities to a strain of *E. coli* isolated from urine sample carrying *bla*_NDM-5_ [55], indicating a rise in the prevalence of MDR *E. coli* strains. Although pNDM-5-0083 closely resembles pCTXM-2271, another multidrug-resistant plasmid found in *E. coli*, it shares common elements such as the mobile insertion sequence *IS91*, the replication initiator *repA*, plasmid replication-associated gene *parA, parB* and IncF plasmid conjugative transfer protein *trbABCDEGIJ*, along with virulence factors like *sitABCD* and *iroBCDEN* involved in iron acquisition. However, pNDM-5-0083 lacks the *floR* resistance gene found in pCTXM-2271 and features a distinct MDR region that sets it apart from pCTXM-2271 [47].

Researchers discovered that the insertion sequences [56, 57], transposons [58] and integrons [59] play key roles in facilitating recombination events and extensive transfer of resistance genes. In pNDM-5-0083, the complex transposon structure *repA-IsI5DIV-InsB-bla*_*TEM1*_*-RmtB-GroEL-IS26-IS30-bla*_*NDM-5*_*-ble*_*MBL*_*-IS91* includes the *repA* element which initiates replication of the MDR region. It is paired with the *pemK/pemI* toxin-antitoxin system, utilizing postsegregational killing to ensure plasmid stability during bacterial transfer [60]. Following this, *IS15DIV*, a member of the *IS6* family similar to *IS26* [61], is inserted, promoting duplications of target site sequences of MDR region alongside an integron *InsB*. This upstream arrangement in the MDR region—*repA-pemK/I-IsI5DIV-InsB-bla*_*TEM1*_*-RmtB*—is analogous to that in the unnamed plasmid A (CP104348.1). while the sequence *IS26-IS30-bla*_*NDM-5*_*-ble*_*MBL*_*-IS91* is consistent with those in pNDM-d2e9 and pLZ135-NDM, highlighting a common strategy for the horizontal transfer and homologous recombination of the resistance genes *bla*_*TEM*,_*bla*_*NDM-5*_, and *ble*_*MBL*_ under antibiotic pressure. This complex genetic architecture underscores the dynamic capability of *IS15DIV*, *IS26*, *IS30* and *IS91* elements to facilitate the spread and evolution of resistance genes across bacterial populations.

In addition, unlike these three homologous plasmids, pNDM-5-0083 contains the transposon *Tn21* and integron *intI1* at the 3’-end of its MDR region. Previous studies have confirmed that *Tn21*, known for harboring integrons like *intI1* that incorporate aminoglycoside and beta-lactam resistance genes, is widespread among both clinical and environmental gram-negative isolates [62–64]. This highlights the crucial roles of *intI1* and *Tn21* as primary vectors in the dissemination of antibiotic resistance genes. The presence of these mobile genetic elements at the terminal end of the MDR region in pNDM-5-0083 suggests they may contribute to frequent homologous recombination events, resulting in a novel variable region compared to its related plasmids. Moreover, another integron *insB* and the molecular chaperone *GroEL* are integrated within the MDR region, enhancing the expression, and ensuring the proper folding of antibiotic resistance proteins, respectively. Together, the strategic incorporation of these elements within the MDR region not only augments the functionality of resistance genes but also plays a pivotal role in enhancing plasmid stability. It promotes the horizontal transfer of the multidrug resistance attributes between different plasmids and the widespread dissemination across various bacterial strains, while ensuring the effective and stable expression of resistance determinants.

In addition to the resistance genes located on both chromosomal and plasmid DNA, mechanisms of antibacterial resistance in *E. coli* often involve virulence factors that promote drug efflux and enhance biofilm formation [30, 31]. Biofilms contribute to antibiotic resistance by creating a protective environment that limits antibiotic penetration and supports the persistence of resistance genes within bacterial communities. In our study, chromosomally encoded virulence genes such as *fdeC*, *yagX/ecpC*, and *vgrG/tssI* were found to enhance bacterial adhesion to host cells, potentially facilitating biofilm formation [65–67]. Moreover, virulence determinants carried by the pNDM-5-0083 plasmid, such as the iron acquisition systems (e.g., the *iro* and *sit* gene families), may indirectly bolster biofilm maintenance by impacting the nutritional status and survival capabilities of the bacteria [68, 69]. While virulence factors like colicin M and enolase do not directly induce biofilm formation, they augment bacterial resilience to environmental stresses, thereby aiding the transmission and endurance of resistance genes [70, 71].

However, our study recognizes certain limitations, particularly the absence of gene editing experiments to confirm the functionality of mobile genetic elements and virulence factors within the plasmid post-annotation. Future research should prioritize experimental studies on biofilm formation to thoroughly investigate the intricate relationships between biofilm development, virulence factors, and antibiotic resistance. This approach will deepen our understanding of the mechanisms that facilitate the persistence and transmission of antibiotic-resistant *E. coli* strains. Additionally, infection control statistics from our institution from 2017 to 2023 have demonstrated a relatively stable CPE isolation rate, fluctuating between 2.5% and 5.4%. Within this dataset, the highest resistance rates for *Klebsiella pneumoniae* against imipenem and meropenem reached 5.8% and 6.8%, respectively, while for *E. coli*, they peaked at 1.2% and 1.4%. Compared to the data from the China Antimicrobial Resistance Surveillance System (CHINET), our institution’s rates of carbapenem-resistant *Klebsiella pneumoniae* are lower, whereas those of carbapenem-resistant *E. coli* align with national averages. Although the newly discovered *bal*_*NDM-5*_-carrying plasmid has not increased the CPE isolation rate at our institution, this study still holds significant value for understanding and managing antibiotic resistance in a broader context.

## Conclusion

This study presents a detailed analysis of pNDM-5-0083, a novel *bla*_*NDM-5*_-carrying plasmid discovered in an *E. coli* isolate. Our comprehensive genomic annotation and analysis shed light on the evolutionary dynamics of MDR region in pNDM-5-0083 and illuminated the genetic elements which might facilitate the genetic recombination to form a unique plasmid architecture. These insights highlight potential targets for combatting the spread of antibiotic resistance, emphasizing the importance of understanding plasmid dynamics in microbial resistance mechanisms.

### Supplementary Information

Supplementary Material 1.

## Data Availability

Sequence data generated for this study have been uploaded in the NCBI GenBank, CP140466 to CP140469. The public data used in this study were CP026201.1, MF353156.1, CP104348.1, MF589339.1, and CP010149.1, respectively.

## Abbreviations

AmpC: AmpC ß-lactamase
BLAST: the basic local alignment search tool
CLSI: Clinical and Laboratory Standards Institute
CPE: carbapenemase-producing *Enterobacteriaceae*
*E. coli*: *Escherichia coli*
ESBL: extended-spectrum ß-lactamase
ICU: intensive care unit
MIC: minimum inhibitory concentration
MLST: multi-locus sequence typing
MTS: minimum inhibitory concentration test strips
*NDM*: *New Delhi metallo-ß-lactamase*
PFGE: pulsed-field gel electrophoresis
