# Supplementary material for: Unveiling the genetic architecture and transmission dynamics of a novel multidrug-resistant plasmid harboring blaNDM-5 in E. Coli ST167: implications for antibiotic resistance management
Source: BMC Microbiol. 2024 May 23;24:178. doi: 10.1186/s12866-024-03333-1 (PMC11112900; doi:10.1186/s12866-024-03333-1)
Supplement: Supplementary file 1 — Supplementary Material 1. [file 12866_2024_3333_MOESM1_ESM.docx]

**Supplementary Figure**

**
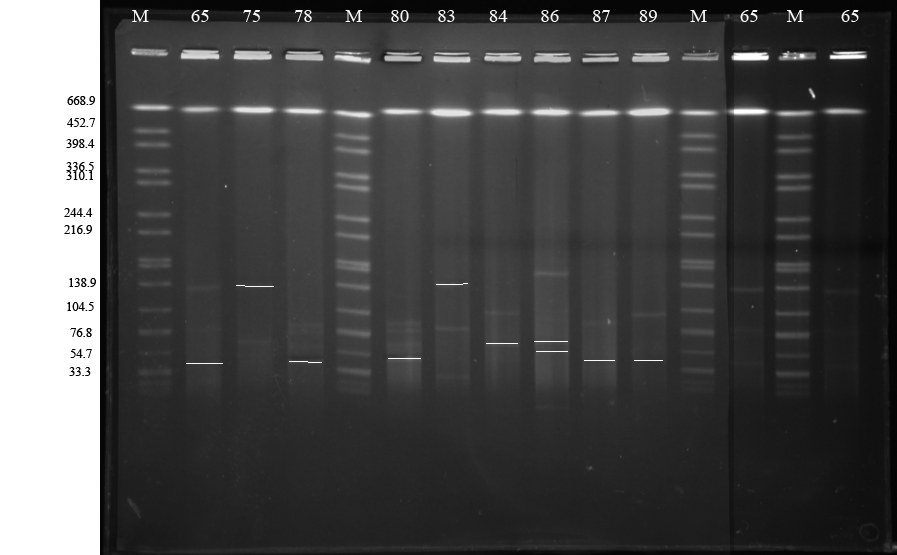
**

**Supplementary Figure 1. The original S1-PFGE gel labeled with *bla*_NDM-5_ DNA probe.** The gel was probed with a *bla_NDM-5_*-specific DNA probe to visualize the presence and location of the *bla_NDM-5_* gene within the plasmid DNA extracted from the isolates. Bands indicating the *bla_NDM-5_* gene are highlighted by detecting the digoxigenin-label. The lane labeled '83' corresponds to the *E. coli* GZ04-0083 isolate, showcasing the specific banding pattern of the *bla_NDM-5_* gene within this sample.


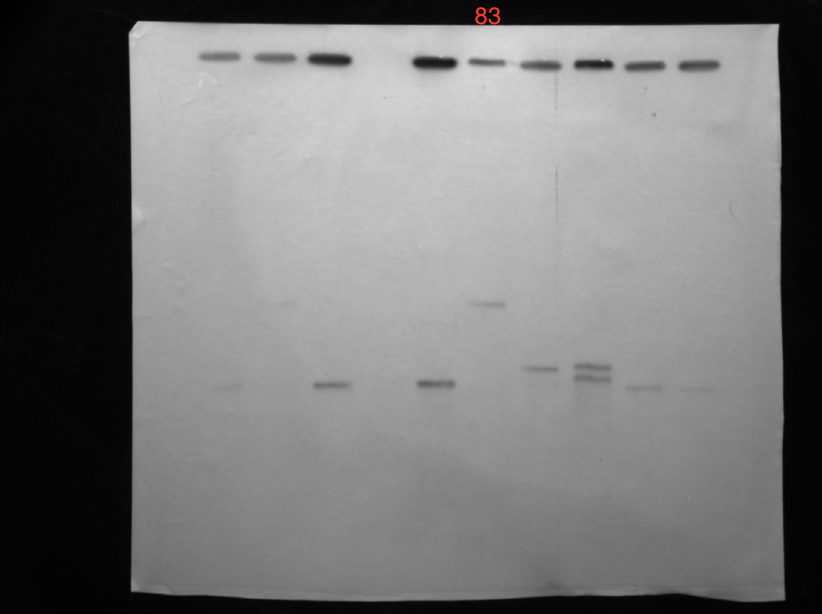


**Supplementary Figure 2. The original Southern blot image specific to *bla_NDM-5_.*** Southern blot analysis targeted at the *bla_NDM-5_* gene illustrated the distribution and intensity of the *bla_NDM-5_* signals across the gel, offering detailed insights into the presence and quantity of the *bla_NDM-5_* gene within the plasmid DNA from the isolates. Notably, the lane labeled '83' showcases the specific banding pattern of the *bla_NDM-5_* gene in the *E. coli* GZ04-0083 isolate, highlighting its distinct genetic profile.
